# The Kinetic Specificity of Plyometric Training: Verbal Cues Revisited

**DOI:** 10.1515/hukin-2015-0122

**Published:** 2015-12-30

**Authors:** Talin Louder, Megan Bressel, Eadric Bressel

**Affiliations:** 1 Biomechanics Laboratory, HPER Department, Utah State University, Logan, UT; 2 Spring Creek Medical Center, Providence, UT

**Keywords:** jumping, agility, biomechanics

## Abstract

Plyometric training is a popular method utilized by strength and conditioning professionals to improve aspects of functional strength. The purpose of this study was to explore the influence of extrinsic verbal cueing on the specificity of jumping movements. Thirteen participants (age: 23.4 ± 1.9 yr, body height: 170.3 ± 15.1 cm, body mass: 70.3 ± 23.8 kg,) performed four types of jumps: a depth jump “as quickly as possible” (DJT), a depth jump “as high as possible” (DJH), a countermovement jump (CMJ), and a squat jump (SJ). Dependent measures, which included measurement of strength and power, were acquired using a force platform. From the results, differences in body-weight normalized peak force (BW) (DJH: 4.3, DJT: 5.6, CMJ: 2.5, SJ: 2.2), time in upward propulsion (s) (DJH: 0.34, DJT: 0.20, CMJ: 0.40, SJ: 0.51), and mean acceleration (m·s-2) (DJH: 26.7, DJT: 36.2, CMJ: 19.8, SJ: 17.3) were observed across all comparisons (p = 0.001 – 0.033). Differences in the body-weight normalized propulsive impulse (BW·s) (DJH: 0.55, DJT: 0.52, CMJ: 0.39, SJ: 0.39) and propulsive power (kW) (DJH: 13.7, DJT: 16.5, CMJ: 11.5, SJ: 12.1) were observed across all comparisons (p = 0.001 – 0.050) except between the CMJ and SJ (p = 0.128 – 0.929). The results highlight key kinetic differences influencing the specificity of plyometric movements and suggest that verbal cues may be used to emphasize the development of reactive strength (e.g. DJT) or high-velocity concentric power (e.g. DJH).

## Introduction

Defined loosely, reactive strength is the ability to react effectively to forces placed on the body by the environment (e.g. ground reaction forces). Typically, this reaction is followed immediately with a coordinated movement utilizing powerful, concentric muscle action. Specialized jumping or plyometrics are exercises that target one’s ability to change quickly from an eccentric to concentric muscle action, commonly referred to as the stretch-shortening cycle (Enoka, 1993). Reactive strength and the stretch-shortening cycle are often defined synonymously (Flanagan et al., 2008). However, one’s ability to react effectively to environmental forces may be considered independent of one’s ability to subsequently produce a powerful concentric movement (Sheppard and Young, 2006), as tasks that require a reaction may not always be followed with ‘explosive’ concentric actions (e.g. drop landings). Additionally, reactive strength should be broadly defined as ‘the ability to react to environmental forces placed on the body, since it is dependent on the integration of multiple biological systems (e.g. neuromuscular) and not specific to the mechanics of the musculotendinous unit (MTU).

Proper execution of plyometric movements is thought to improve the development of reactive strength and high-velocity concentric power (Sheppard and Young, 2006). Moreover, plyometric training may assist with injury prevention in various competitive sports. Accordingly, plyometric training continues to receive a high level of interest among researchers, coaches and athletes. Continued interest in plyometric research is due, in part, to the need to advance consensus regarding its role in improving physical performance and injury prevention (Hill and Leiszler, 2011).

Specificity, a key training principle, suggests that movements performed in training should elicit an overload stimulus that explicitly improves the performance of sport-specific movement tasks (Baechle and Earle, 2000). The specificity of certain training modes, such as resistance training, is fairly straightforward and based on key program design characteristics such as intensity, volume, frequency and periodization (Baechle and Earle, 2000). While these program characteristics are indeed important for a plyometric training program design (Jensen and Ebben, 2007), differentiating the intensity of various plyometric type movements is more complex.

Previously, plyometric type movements have been classified through the use of subjective classifications such as “high” and “low” intensity under the presumption that a higher intensity movement corresponds to greater stresses placed on the tissues of the body (Baechle and Earle, 2000). Recent interest of the plyometric literature has been focused on disbanding from the subjective classification of plyometric-type movements in favor of a kinetic-based (e.g. force-time, power) approach for assessing the intensity and specificity of various plyometric exercises (Jensen and Ebben, 2007; Ebben et al., 2011; Jidovtseff et al., 2014; Van Lieshout et al., 2014).

Prior research has indicated that verbal cues influence the specificity and manipulability of various plyometric type movements. Young et al. (1995) originally visited this concept using verbal instruction to perform the drop jump to achieve maximum height or minimal contact time. As could be expected, subjects jumped higher when instructed to jump as high as possible and produced lower contact times with the ground when instructed to minimize ground contact time (Young et al., 1995).

More recently, Jidovtseff et al. (2014) examined how the combined use of extrinsic (interaction with the environment; e.g. contact time / jump height) and intrinsic (relating to the body’s movement; e.g. knee flexion) verbal cueing influenced kinetic force platform measures across eight unique jumping variations. Differences in kinetic measures (e.g. displacement, velocity, power) were observed, depending on the application of specific cues. However, from their results it was not clear whether changes in these parameters were elicited from the use of extrinsic cuing (e.g. “minimal contact time”, “jump as high as possible”), intrinsic cuing (e.g. “little / deep knee flexion”), or the cues in combination. Prior research has indicated that the type of cueing (intrinsic or extrinsic) influences muscular force production, as greater forces were observed when subjects’ attention was directed extrinsically (Marchant et al., 2009). Therefore, further research is appropriate to determine the influence of different types of verbal cueing (e.g. extrinsic or intrinsic) on kinetic measures of plyometric performance.

The purpose of the present study was to evaluate the specificity and manipulability of commonly performed plyometric movements by quantifying kinetic characteristics in male and female subjects when utilizing extrinsic verbal cueing. The study sought to identify whether simple extrinsic cues could be an effective tool for targeting the development of certain components of functional strength, including the development of reactive strength and concentric muscle power.

## Material and Methods

### Participants

Thirteen recreationally active young adults (Males = 8, Females = 5) were asked to volunteer as subjects (age: 23.4 ± 1.9 yr, body height: 170.3 ± 15.1 cm, body mass: 70.3 ± 23.8 kg). Subjects were recruited from university intramurals and were excluded if they presented a lower extremity injury or history of injury 6 months prior to the study. Subjects were required to sign an informed consent form approved by the Utah State University Institutional Review Board. There was no subject attrition for the duration of the study.

### Measures

Using methods described previously (Enoka, 1993), raw force platform data (1000 Hz, Threshold: 25 N) was used to compute the following dependent measures: body-weight normalized maximum force (BW), time in upward propulsion (s), propulsive impulse (BW·s), max propulsive power (W), and mean acceleration (m·s^−2^).

### Procedures

Each subject performed, in random order, four common jump variations. All jumps were performed on a force platform (Bertec Corporation, Columbus, OH) connected to a PC (Dell Inc., Round Rock, TX). The countermovement jump condition (CMJ) required subjects to jump as high as possible from the ground. The CMJ was accomplished through the utilization of a short eccentric phase followed by a concentric action, driving the whole body center of gravity upwards. The squat jump condition (SJ) emphasized the concentric phase, as it required subjects to squat and jump as high as possible in a single, fluent motion. Two depth jump conditions were performed from a height of 0.35 m (Van Lieshout et al., 2014). For the depth jump conditions, subjects were asked to ‘step forward’ off the box onto a force platform, followed by a subsequent vertical jump with verbal cues to jump ‘as high as possible’ (DJH) or ‘as quick as possible’ (DJT). All subjects received instruction from the same researcher. Subjects were given demonstrations of all conditions and an unrestricted amount of practice repetitions prior to the measured trial for familiarization. No subject performed more than five practice jumps per condition. Each trial was collected for 15 s and was manually triggered and recorded using AcqKnowledge software (Biopac Systems, Inc., Goleta, CA).

### Analysis

Body weight was computed by averaging force data across a 5 s static trial (standing on the force platform) for each subject. Body-weight normalized max force was calculated as the greatest force value during a take-off divided by body weight in Newtons. Time in upward propulsion was computed as the length of time the force time-series stayed at or above subjects’ body weight (time in upward propulsion) during the jumping movement. Since vertical ground reaction forces above body weight signify a positive acceleration of the body upwards, the propulsive impulse was calculated by integrating the fragment of force time-series above body weight. Endpoints for this data corresponded to body weight (computed from the static trials) and were obtained by linear interpolation. Propulsive power at every time point *t* during upward acceleration was computed by setting initial velocity to zero and applying the following formula:

1) $$Power_{t}=F_{t}v_{t}=F_{t}\left(\int_{0}^{t}at\right)$$

Since initial velocity is purposefully set to zero, it should be noted that this measure of propulsive power is a constant overestimation of the true power of the body moving through space. While it is an overestimation, the benefit of this analysis is that it factors out external work and provides insight into the work performed by the body on the environment. This measure of power corresponds to the segment of force time-series wherein acceleration of the body is positive, or propulsive. Max propulsive power was obtained by using the greatest value across time points during this propulsive phase.

Lastly, mean acceleration was computed by multiplying the body weight-normalized force data (F_BW_) by 9.8 followed by averaging across all data points.

### Data Sectioning

Previous research has sought to quantify the intensity of various jumping movements by examining ground reaction and joint reaction forces (Jensen and Ebben, 2007; Ebben et al., 2011; Jidovtseff et al., 2014). This research generally focused on sectioning force plate data into eccentric and concentric phases (Jidovtseff et al., 2014). This sectioning procedure entails double integration of the acceleration time series. Therefore, identification of the transition from eccentric to concentric is based on estimated center of gravity displacement using the assumption of a perfectly elastic collision between the feet and force platform. This method of sectioning is subject to error if energy is dissipated within the system (e.g. body tissues). An alternative method used in the present study is to isolate and make inferences on force plate data that are propulsive; or above body weight, as this provides insight into the work performed by the body on the environment.

Differences in dependent measures were assessed using 2 (gender) × 4 (jump type) ANOVA (*α =* 0.05). For any significant effects on the jump type, pairwise comparisons were obtained across conditions using the Bonferroni post-hoc assessment. Cohen’s *d* effect sizes (ES) were computed to appreciate the meaningfulness of any significant differences Cohen, 1988).

## Results

### Max Force (Acceleration)

There was a significant main effect for the jump type (*F* = 44.4, *p* < 0.001), but no effect for gender (*p* = 0.569) or the interaction between gender and the jump type (*p* = 0.743). Pairwise comparisons revealed significant differences (*p* < 0.010) across all jump types (Table 1). Effect sizes across jump types ranged from 0.77 to 2.72.

### Time in Propulsion

There was a significant main effect for the jump type (*F* = 43.3, *p* < 0.001), but no effect for gender (*p* = 0.352) or the interaction between gender and the jump type (*p* = 0.826). Pairwise comparisons revealed significant differences (*p* < 0.030) across all jump types (Table 1). Effect sizes across jump types ranged from 0.65 to 3.36.

### Propulsive Impulse

There was a significant main effect for the jump type (*F* = 82.1, *p* < 0.001) and gender (*F* = 17.3, *p* = 0.002, Male = 0.49 ± 0.05 BW·s, Female = 0.42 ± 0.03 BW·s), but no interaction between gender and the jump type (*p* = 0.349). Pairwise comparisons revealed significant differences (*p* < 0.002, Table 1) across all jump types except between the CMJ and SJ conditions (*p* = 0.929, ES = 0.12). Effect sizes across the statistically different jump types ranged from 0.53 to 2.76.

### Max Propulsive Power

Prior to statistical analysis, propulsive power was normalized to body mass (kW/kg). There was a significant main effect for the jump type (*F* = 32.4, *p* = 0.018) and gender (*F* = 34.3, *p* < 0.001, Male = 0.20 ± 0.02 kW/kg, Female = 0.16 ± 0.02 kW/kg), but no interaction between gender and the jump type (*p* = 0.187). Pairwise comparisons revealed significant differences (*p* < 0.004, Table 1) across all jump types except between the CMJ and SJ conditions (*p* = 0.111, ES = 0.24). Effect sizes across the statistically different jump types ranged from 0.78 to 2.13.

### Mean Acceleration

There was a significant main effect for jump types (*F* = 61.7, *p* < 0.001), but no effect for gender (*p* = 0.438) or the interaction between gender and the jump type (*p* = 0.917). Pairwise comparisons revealed significant differences (*p* < 0.001) across all jump types (Table 1). Effect sizes across jump types ranged from 0.94 to 3.34.

## Discussion

The results, similar to the findings of Jidovtseff et al. (2014), demonstrate how simple, extrinsic verbal cues can significantly impact the kinetic specificity of plyometric-type movements. Based on kinetic characteristics (e.g. increased max F / mean acceleration, increased impulse, increased power, and decreased contact time), the results of the present study indicate that reactive strength may best be targeted by performing depth jumps using verbal cues that emphasize minimal contact time. Kinetic data indicate the opposite for the squat jump (e.g. decreased max F / mean acceleration, decreased impulse, decreased power, and increased contact time), which may be best for targeting high-velocity concentric action. Results for the DJH and CMJ fit between what was observed for the DJT and SJ (Table 1). Results suggest that the DJH likely targeted reactive strength to a greater degree compared to the CMJ.

The plyometric literature has established the importance of varying plyometric training exercises to target both eccentric and high-velocity concentric muscular actions, suggesting that verbal cues used in the present study may provide an added performance benefit (de Villarreal et al., 2009). Moreover, despite some ambiguity (Goodall et al., 2013; Pfeiffer et al., 2006; Stevenson et al., 2014), plyometric training is clinically effective in conditioning the body to accept large accelerations and protect the integrity of tissues and joint structures (Bien, 2011; Stojanovic and Ostojic, 2012; Sugimoto et al., 2013; Young et al., 2001). It is plausible that inconsistencies (e.g. uncertain efficacy in the prevention of ACL injury) observed in prior research are due, in part, to the specificity of exercise protocols used.

**Figure 1:**
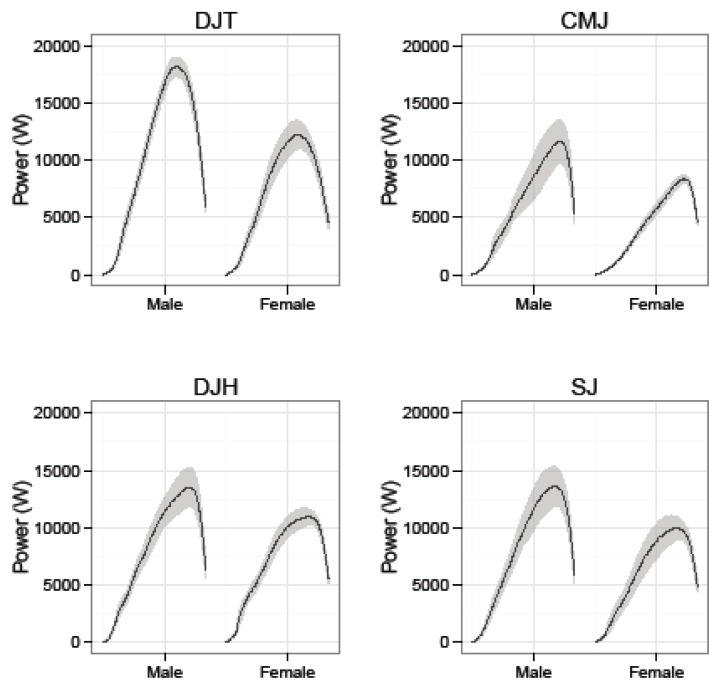
Ensemble averages ± SE for propulsive power (W)

**Figure 2:**
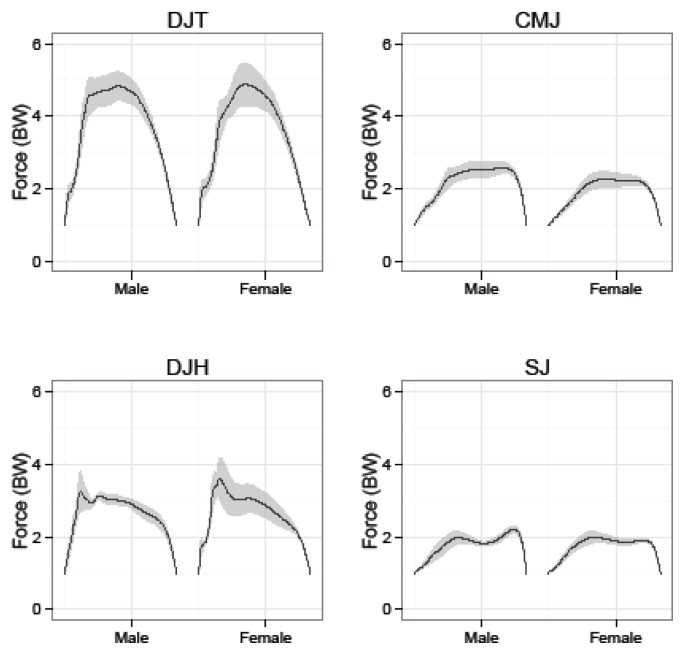
Ensemble averages ± SE for force (BW)

While a comparison on gender was not a main focus of the present study, previous research documents differences in the kinetic specificity of jumping movements across gender. The original aspects of our data identified gender differences in the body mass normalized propulsive impulse and max propulsive power (Figure 1). These findings were not mirrored by differences in parameters of the force time series (e.g. max force (peak acceleration), time in propulsion, (Figure 2) or mean acceleration. In other words, we observed gender differences for measures computed using integral calculus and force platform data, but not for measures taken directly from the force time series. As evidenced by Laffaye and Wagner (2013), differences in integrated force platform data may be due to a greater eccentric rate of force development in males (+11.6%) than in females. This measure was not included in the present study. Additionally, it should be noted that the gender comparison was conducted utilizing small samples, a limitation of the present study. Therefore, it is important to sensibly interpret these findings, given the low statistical power likely to result from the small sample sizes used.

Furthermore, electromyography (EMG) results from Ebben et al. (2008) showed a reduction in motor unit activity for depth jumps compared to other jumping movements, suggesting increased reliance on passive force development. This supports our results and suggests that depth jumps emphasize the eccentric action of involved musculature to a greater degree than other concentric-dominant movements. This does not suggest, however, that effective jump training programs should target either eccentric or concentric-dominant movements. The plyometric literature provides strong evidence that eccentric and concentric actions act jointly in producing functional movements, suggesting the need to address both muscular actions in a program design (Foure et al., 2011; Laffaye and Wagner, 2013). This is further supported by a meta-analysis by de Villareal et al. (2009) who observed that programs emphasizing eccentric and concentric actions of the musculature (e.g. depth jumps and squat jumps) were superior to programs emphasizing either action independently.

It can be observed from the results of the present study that the kinetics of common plyometric-type movements may be manipulated using simple extrinsic verbal cueing which, in turn, could be utilized to enhance the specificity of plyometric training. These results, similar to those reported previously (Jidovtseff et al., 2014), are relevant to strength and conditioning and clinical professionals as they highlight how extrinsic verbal cues affect the kinetic specificity of plyometric-type movements. The clinical relevance of these observations is that professionals may potentially utilize extrinsic cues to better target the development of various components of functional strength including reactive strength and concentric muscle power.

## Figures and Tables

**Table 1:** Pairwise comparisons across all jump

Dependent Measure		DJT	DJH	CMJ	SJ
Max Force (BW)	Mean	5.58	4.33^a^	2.51^a^,^b^	2.22^a^,^b^,^c^
	SD	1.24	1.04	0.38	0.30
Contact Time (s)	Mean	0.20	0.34^a^	0.40^a^,^b^	0.51^a^,^b^,^c^
	SD	0.05	0.07	0.09	0.09
Impulse (BW·s)	Mean	0.52	0.55^a^	0.39^a^,^b^	0.39^a^,^b^
	SD	0.06	0.04	0.06	0.07
Mean Acc (m·s^−2^)	Mean	36.17	26.69^a^	19.76^a^,^b^	17.34^a^,^b^,^c^
	SD	5.63	4.54	2.56	2.14
Power (kW·kg^−1^)	Mean	0.23	0.19^a^	0.16^a^,^b^	0.17^a^,^b^
	SD	0.03	0.02	0.03	0.03

a Statistically different from the DJT.

b Statistically different from the DJH.

c Statistically different from the CMJ.
